# Thrombin enhances the adhesion and migration of human colon adenocarcinoma cells via increased beta 3-integrin expression on the tumour cell surface and their inhibition by the snake venom peptide, rhodostomin.

**DOI:** 10.1038/bjc.1996.161

**Published:** 1996-04

**Authors:** H. S. Chiang, R. S. Yang, T. F. Huang

**Affiliations:** Pharmacological Institute, College of Medicine, National Taiwan University, Taipei.

## Abstract

The interactions between tumour cells and the microvasculature, including the adhesion of tumour cells to endothelium and extracellular matrix (ECM) as well as their migratory ability, are prerequisites for metastasis to occur. In this study we showed that thrombin is capable of enhancing in vitro tumour cell metastatic potential in terms of adhesive properties and migratory response. Following exposure to subclotting concentrations of thrombin, SW-480 human colon adenocarcinoma cells exhibited increased adhesion to both the endothelium and ECM component (i.e. fibronectin). Likewise, the pretreatment of thrombin enhanced the migratory ability of SW-480 cells. The enhanced adhesion was significantly inhibited by complexing of thrombin with its inhibitor hirudin, or by serine proteinase inhibition with 3,4-DCI, but was unaffected by pretreatment of tumour cells with actinomycin D or cycloheximide. The effect of thrombin resulted in an upregulated cell-surface expression of beta 3 integrins, a group of receptors mediating interactions between tumour cells and endothelial cells, and between tumour cells and ECM. Antibodies against beta 3 integrins effectively blocked both the enhanced adhesion and migration. This thrombin-mediated up-regulation of beta 3 integrins involved the activation of protein kinase C (PKC) as thrombin-enhanced adhesion was diminished by PKC inhibition. Rhodostomin, an Arg-Gly-Asp-containing antiplatelet snake venom peptide that antagonises the binding of ECM toward beta 3 integrins on SW-480 cells, was about 600 and 500 times, more potent that RGDS in inhibiting thrombin-enhanced adhesion and migration respectively. Our data suggest that PKC inhibitors as well as rhodostomin may serve as inhibitory agents in the prevention of thrombin-enhanced metastasis.


British Journal of Cancer (1996) 73, 902-908
© 1996 Stockton Press All rights reserved 0007-0920/96 $12.00

### Thrombin enhances the adhesion and migration of human colon adenocarcinoma cells via increased $\beta_3$-integrin expression on the tumour cell surface and their inhibition by the snake venom peptide, rhodostomin

H-S Chiang[1], R-S Yang[2] and T-F Huang[1]

[1]*Pharmacological Institute and* [2]*Department of Orthopaedics, College of Medicine, National Taiwan University, Taipei, Taiwan.*

**Summary** The interactions between tumour cells and the microvasculature, including the adhesion of tumour cells to endothelium and extracellular matrix (ECM) as well as their migratory ability, are prerequisites for metastasis to occur. In this study we showed that thrombin is capable of enhancing *in vitro* tumour cell metastatic potential in terms of adhesive properties and migratory response. Following exposure to subclotting concentrations of thrombin, SW-480 human colon adenocarcinoma cells exhibited increased adhesion to both the endothelium and ECM component (i.e. fibronectin). Likewise, the pretreatment of thrombin enhanced the migratory ability of SW-480 cells. The enhanced adhesion was significantly inhibited by complexing of thrombin with its inhibitor hirudin, or by serine proteinase inhibition with 3,4-DCI, but was unaffected by pretreatment of tumour cells with actinomycin D or cycloheximide. The effect of thrombin resulted in an up-regulated cell-surface expression of $\beta_3$ integrins, a group of receptors mediating interactions between tumour cells and endothelial cells, and between tumour cells and ECM. Antibodies against $\beta_3$ integrins effectively blocked both the enhanced adhesion and migration. This thrombin-mediated up-regulation of $\beta_3$ integrins involved the activation of protein kinase C (PKC) as thrombin-enhanced adhesion was diminished by PKC inhibition. Rhodostomin, an Arg-Gly-Asp-containing antiplatelet snake venom peptide that antagonises the binding of ECM toward $\beta_3$ integrins on SW-480 cells, was about 600 and 500 times, more potent than RGDS in inhibiting thrombin-enhanced adhesion and migration respectively. Our data suggest that PKC inhibitors as well as rhodostomin may serve as inhibitory agents in the prevention of thrombin-enhanced metastasis.

**Keywords:** thrombin; metastasis; $\beta_3$ integrin; protein kinase C; Arg-Gly-Asp containing peptide

The metastatic cascade of tumour cells is a sequence of complex events characterised by their growth at the primary site, invasion of surrounding tissues, penetration into vasculature and transport to distant sites and implantation and formation of secondary lesions. The haematogenous phase of metastasis involved a variety of cell–cell and cell–ECM interactions. Tumour cell interaction with endothelium and ECM, a process believed to determine organ-specific metastasis, is mediated by a wide spectrum of cell-surface adhesion molecules, including integrin receptors (Honn *et al.*, 1992*a*, *b*). There is evidence that systemic or local activation of blood coagulation promotes metastasis, whereas inhibition of the blood clotting cascade favours the host and diminishes metastatic spread (Honn and Sloane, 1984). However, the exact nature of this association and its potential practical significance are not yet completely understood.

It has been known for over a century that most cancer patients manifest laboratory signs of hypercoagulability and some develop thromboembolic disease (Rickles *et al.*, 1992). Clinical trials of anticoagulants or fibrinolytic agents have exhibited improvements in the incidence of tumour regression and in overall survival (Zacharski *et al.*, 1992*a*). Therefore, the mainstream of clinical and basic investigators has focused on the role of interactions between tumour cells and the host coagulation system. In certain tumour types, *in situ* fibrin, the final product of blood coagulation, is present predominantly at the host–tumour interface in abundant amounts (Zacharski *et al.*, 1992*b*). Fibrin deposited at the primary site constitutes a component of tumour stroma, which protects tumour cells against cells of the host defence system and facilitates tumour angiogenesis (Zacharski *et al.*, 1992*b*; Costantini and Zacharski, 1993). Thrombin, a pluripotent bioregulatory serine proteinase, was reported to enhance the metastatic phenotype of mammary tumour cells by increasing their proliferative response (Medrano *et al.*, 1987) and was also found to be a potent mitogen for tumour cells (Bruhn and Zurborn, 1983). In fact, in our previous study (Chiang *et al.*, 1994*a*), thrombin was shown to mediate SW-480 tumour cell-induced platelet aggregation (TCIPA), which might be important for successful metastasis to occur (Cavanaugh *et al.*, 1988).

In recent years, purified antiplatelet components from snake venoms, including trigramin-like antiplatelet peptides (Huang *et al.*, 1987*a*, 1991*a*, *b*; Rucinski *et al.*, 1990; Shebuski *et al.*, 1989) have been widely studied. Trigramin, an Arg-Gly-Asp (RGD)-containing peptide purified from venom of the snake *Trimeresurus gramineus*, is a specific antagonist of platelet membrane glycoprotein IIb/IIIa (Huang *et al.*, 1987*a*, 1989). Rhodostomin, an RGD-containing peptide purified from the venom of the Malayan pit viper, *Agkistrodon rhodostoma*, likewise directly impairs fibrinogen interaction with its specific receptor associated with glycoprotein IIb/IIIa (Huang *et al.*, 1987*b*, 1990). These trigramin-like peptides all contain an RGD epitope, are rich in cysteine and bind with high affinity to the surface of platelets.

The present study documents the effect of thrombin treatment with SW-480 human colon adenocarcinoma cells on regulation of their surface integrin expression, cell adhesive and migratory properties. Rhodostomin was found to strongly inhibit thrombin-enhanced tumour cell adhesion and migration. We also compared the effect of MAbs and synthetic peptide RGDS.

### Materials and methods

### *Materials*

SW-480 human colon adenocarcinoma cells were provided by the Department of Bacteriology, College of Medicine, National Taiwan University. *Agkistrodon rhodostoma* (or *Calloselasma rhodostoma*) venom was purchased from Latoxan (France) and stored at −20°C. Rhodostomin was purified from venom of *A. rhodostoma* as previously

Correspondence: T-F Huang, Pharmacological Institute, College of Medicine, National Taiwan University, No. 1, Sec. 1, Jen-Ai Rd., Taipei, Taiwan.
Received 5 July 1995; revised 10 October 1995; accepted 27 October 1995

described (Huang *et al*., 1990). Synthetic peptide RGDS was purchased from Peninsula Laboratories, CA, USA. Human thrombin (3000 NIH units $mg^{-1}$), hirudin (grade IV from leeches), 3,4-dichloroisocoumarin (3,4-DCI), cycloheximide, actinomycin D and fibronectin (from bovine plasma) were obtained from Sigma, St Louis, MO, USA. Thrombin–hirudin or thrombin–serine protease inhibitor complex was formed by incubation for 30 min at 37°C of equimolar concentrations of human thrombin with either inhibitor (heparin, 5 U $ml^{-1}$; 3,4-DCI, 0.1 mM). The thrombin–hirudin complex had no fibrinogen clotting activity. Staurosporine was obtained from Biomol Research Laboratories, PA, USA. Calphostin C (isolated from *Cladosporium cladosporioides*) was from Research Biochemicals International, MA, USA. Goat anti-mouse IgG-FITC was from Boehringer, Mannheim, Germany. Monoclonal antibodies (MAbs) $7E_3$ and $10E_5$ against platelet GPIIb/IIIa complex were kindly supplied by Dr B Coller (State University of New York, Stony Brook, USA). The specificity of these antibodies for human platelet $\alpha_{IIb}\beta_3$ was reported previously (Grossi *et al*., 1988; Chopra *et al*., 1988). MAb against $\beta_1$ integrin (MAB1977) was obtained from Chemicon, CA, USA MCA698 (anti-$\alpha_5\beta_1$) and MCA699 (anti-$\alpha_6\beta_1$) were purchased from Serotec, Bicester, UK.

### *Methods*

*Cell culture* SW-480 human colon adenocarcinoma cells were grown in 95% air–5% carbon dioxide in Dulbecco's modified Eagle medium (DMEM) tissue culture medium supplemented with 10% heat-inactivated fetal calf serum (FCS), 2 mM glutamine, penicillin (100 U $ml^{-1}$) and streptomycin (100 $\mu g$ $ml^{-1}$). Cells were passed and harvested for experiments before reaching confluence. Human umbilical vein endothelial cells (HUVECs) were prepared as described by Jaffe *et al*, (1973). Umbilical cord veins were cannulated and flushed with cord buffer to remove blood and then filled with 0.1% collagenase solution (type I, Sigma) for 15–20 min at 37°C. Detached cells were recovered by flushing with M199 and collected by centrifugation at 1000 r.p.m. for 5 min. The cells were then suspended in M199 containing 20% FCS, penicillin (100 U $ml^{-1}$) and streptomycin (100 $\mu g$ $ml^{-1}$) before seeding. After 18–24 h incubation the medium was removed, the cells washed gently before fresh medium was added. Human microvascular endothelial cells (HMVECs) were kindly provided by Dr MW Swaim (Duke University Medical Center, NC, USA) and grown in DMEM-F12 medium containing 20% FCS. HMVEC line was used within sixth passages. Both endothelial cells were identified as positively immunofluorescent staining for von Willebrand factor antigen.

*Adhesion studies* SW-480 cells were harvested with 0.25 mM EDTA (5 min, 37°C). EDTA has been demonstrated to dissociate the platelet IIb/IIIa complex (Fitzgerald and Phillips, 1985). Therefore, tumour cells were washed free of serum proteins with Hanks' balanced salt solution (HBSS, pH 7.25) containing 2 mM $Ca^{2+}$ and 2 mM $Mg^{2+}$. 2′,7′-Bis-(2-carboxyethyl)-5(and-6)-carboxyfluorescein acetoxymethyl (BCECF-AM, Molecular Probes, OR, USA) has been used in fluorescence-based viability assessment in adherent cell cultures (Vaporciyan *et al*., 1993). In our study, cells ($5 \times 10^4$) were incubated with fluorescent dye (2 $\mu g$ $ml^{-1}$) in HBSS for 30 min at 37°C. Following incubation, cells were washed once in phosphate-buffered saline (PBS), and finally resuspended in HBSS containing 2 mM $Ca^{2+}$ and 2 mM $Mg^{2+}$. Monolayers of endothelial cells, grown to confluence in Costar 48-well plates, were used for adhesion studies. Aliquots of fibronectin (1.5 $\mu g$ per well) dissolved in PBS were placed in Costar 96-well plates. The coated wells were kept in a laminar flow hood to air dry overnight, and washed with PBS immediately before adhesion assay. Cells were incubated with buffer or 0.5 U $ml^{-1}$ thrombin for 30 min, followed by washing and then addition of buffer, antibodies, RGDS or rhodostomin and incubated for 30 min before adhesion assay. In some experiments, cells were treated with various concentrations of cycloheximide, actinomycin D, staurosporine or calphostin C for 20 min at room temperature before incubation with thrombin. Pretreated cells were then washed once with PBS and used immediately in adhesion assays. Treated or untreated cells ($2.5 \times 10^4$) were added to each well and incubated (60 min) at 37°C. Each condition was run in quadruplicate and all experiments were repeated at least four times with similar results. Non-adherent cells were removed by aspiration and plates were read with a CytoFluor 2300 fluorescence plate reader (Millipore, Bedford, MA, USA).

*Cell migration assays* Cell migration assays were carried out in millicell-PCF inserts (Millipore) by using standard 24-well tissue culture plates (Costar, USA). Briefly, SW-480 cells were harvested and incubated with BCECF-AM as previously described (Chiang *et al*., 1994*b*). The fluorescence-loaded cells were then resuspended in DMEM without serum and preincubated with buffer or thrombin (0.5 U $ml^{-1}$) for 30 min at 37°C, followed by washing and addition of either buffer or 3.5 $\mu g$ $ml^{-1}$ rhodostomin before migration assays. They were added ($3 \times 10^5$ cells $ml^{-1}$) to the upper compartment of a millicell-PCF insert and the lower compartment was filled with medium containing a chemoattractant: fibronectin (30 $\mu g$ $ml^{-1}$) and control medium (serum-free DMEM containing 0.1% bovine serum albumin). The two compartments of the millicell-PCF inserts were separated by a polycarbonate filter (12 $\mu m$ pore size) precoated with gelatin (5 $\mu g$ $ml^{-1}$, Sigma). SW-480 cells were allowed to migrate for 14 h at 37°C in a humidified atmosphere containing 5% carbon dioxide. Cells on the upper side of the filter were removed mechanically, and the adherent cells on the lower side of the filter were read with a CytoFluor 2300 fluorescence plate reader (Millipore Corp.) as described in cell adhesion assays.

*Flow cytometric analysis* Flow cytometric studies were performed to quantify surface expression of integrins (Chiang *et al*., 1994*b*). SW-480 cells were detached (using 0.5 mM EDTA), washed and then stimulated with 0.5 U $ml^{-1}$ thrombin at 37°C for 30 min in 500 $\mu l$ of PBS containing $10^6$ cells per sample. Following washing the cells were fixed with 2.7% paraformaldehyde for 10 min, blocked with normal goat serum (1:2) for 25 min, and labelled with MAbs (20 $\mu g$ $ml^{-1}$) for 1 h. After washing cells were relabelled with goat anti-mouse IgG-FITC. FITC signals were detected and digitised in logarithmic configuration and the data collected on a EPICS computer system. Data were collected in 256-channel resolution and 10 000 cells were counted per experimental group. Fluorescence intensity was directly proportional to the fluorescein label present on the tumour cell surface. All experiments were repeated at least four times.

### Results

### *Effect of thrombin on SW-480 tumour cell adhesion to human endothelial cells*

In this study we showed that SW-480 human colon adenocarcinoma cells respond to human thrombin by increased adhesion to human endothelial cell monolayers, HMVEC and HUVEC. We used different concentrations of thrombin, ranging from 0.01 to 5 U $ml^{-1}$ with incubation time of 30 min. Figure 1a shows that stimulation of SW-480 cells with various concentrations of thrombin exhibited bell-shaped dose–response curves in increasing adhesion to endothelial cells (ECs) with a peak at 0.5 U $ml^{-1}$. The maximal effects for HUVEC and HMVEC were approximately 3.5- and 2.3-fold increases respectively in the number of adherent cells. Both increments were significant effects ($P<0.001$). A concentration of thrombin as low as 0.01 U $ml^{-1}$ enhanced adhesion, whereas higher concentrations (i.e. 5 U $ml^{-1}$) were ineffective.

*Effect of thrombin on SW-480 tumour cell adhesion to fibronectin*

Fibronectin (FN), which is found in the fibro-connective tissue stoma, was also used as a substratum for studies of SW-480 cell-adhesion. Thrombin treatment resulted in a maximal response of approximately 2.5-fold increase in the number of adherent cells. The dose–response curve was also bell-shaped, with the maximal effect at 0.5 U $ml^{-1}$ (Figure 1a). However, similar to the results from adhesion to ECs, subclotting concentrations of thrombin (i.e., 0.01 U $ml^{-1}$) also enhanced adhesion whereas higher concentrations (i.e., 5 U $ml^{-1}$) did not. Incubation of SW-480 cells with thrombin for different time periods also revealed a bell-shaped pattern (Figure 1b). A slight increase was observed 5 min after pretreatment with thrombin and the optimal response occurred when tumour cells were stimulated with thrombin for a period ranging between 30 min and 4 h. Prolonged incubation of tumour cells (8–24 h) with thrombin resulted in a decrease in tumour cell adhesion.

When thrombin was mixed with hirudin, a specific thrombin inhibitor, a profound inhibition was found compared with the buffer control (Figure 2). This suggests that an intact thrombin molecule is apparently required to enhance SW-480 cell adhesion. In addition, an irreversible serine protease inhibitor, 3,4-DCI, was observed to completely block thrombin-enhanced adhesion of SW-480 cells to FN, further suggesting that the active site serine residue is required for the enhancing effect of thrombin. However, thrombin-enhanced adhesion was not affected by pretreatment of cells with either cycloheximide or actinomycin D, the inhibitors of protein synthesis and RNA synthesis respectively (Figure 2).

*Effect of protein kinase C inhibitors on thrombin-enhanced SW-480 tumour cell adhesion to fibronectin*

It is well recognised that protein kinase C (PKC) activation, an important part of signal transduction, is involved in growth regulation of many tumours and in metastasis (Liu *et al.*, 1992). In our previous study, we found that PKC activation resulted in up-regulation of integrin on SW-480 cells and thus increased their adhesion (Chiang *et al.*, 1994*b*). Thrombin was reported to either activate or down-regulate PKC, depending on the cell type studied (Gomez *et al.*, 1988). Therefore we used two PKC inhibitors to examine whether PKC is implicated in thrombin-enhanced SW-480 tumour cell adhesion. Pretreatment of SW-480 cells with either staurosporine (Figure 3a), a potent but relatively less selective PKC inhibitor, or calphostin C (Figure 3b), a selective PKC inhibitor, blocked thrombin-enhanced adhesion to FN in a dose-dependent manner. Maximal inhibition was approximately 80% of basal (i.e. unstimulated) adhesion by either inhibitor, suggesting that PKC activation is a key event in thrombin-enhanced adhesion. However, basal adhesion was not significantly affected by PKC inhibition.

*Effect of rhodostomin and an MAb against $\beta_3$ integrin on thrombin-enhanced SW-480 tumour cell adhesion*

Tumour cell adhesion to ECs and ECM is mediated by cell-surface adhesion molecules, including the integrin receptors. The $\beta_3$ integrins (i.e. $\alpha_{IIb}\beta_3$ and $\alpha_v\beta_3$) were detected in several tumour cells (Chopra *et al.*, 1988; McGregor *et al.*, 1989) that have been implicated in tumour cell–platelet (Chopra *et al.*, 1988), tumour cell–ECM and tumour cell–ECs (Grossi *et al.*, 1989) interactions. Therefore we investigated the possible role of $\beta_3$ integrin in thrombin-enhanced SW-480 cell adhesion. Stimulation of SW-480 cells with 0.5 U $ml^{-1}$ thrombin (30 min at 37°C) was followed by an incubation with $10E_5$ (30 $\mu g$ $ml^{-1}$), an MAb that does not cross-react

**Figure 1** Dose- and time effect of thrombin in enhancing tumour cell adhesion. (**a**) SW-480 cells were pretreated with various concentrations of thrombin for 30 min at 37°C, and tested for adhesion to human endothelial cells (HUVEC, ▲; HMVEC, ●) and fibronectin (□) after 60 min. The adherent cells were evaluated as described in Materials and methods. (**b**) SW-480 cells were preincubated with thrombin (0.5 U $ml^{-1}$) at 37°C for various indicated time intervals and adhesion to fibronectin was assayed. Data are presented as mean ± s.e.m. ($n=4$). $^*P<0.05$, $^{**}P<0.01$, $^{***}P<0.001$.

**Figure 2** Effect of hirudin as well as inhibitors of serine proteinase, protein synthesis and RNA synthesis on thrombin-enhanced SW-480 tumour cell adhesion to fibronectin. SW-480 cells were stimulated with optimal dose of human thrombin (0.5 U $ml^{-1}$), thrombin–hirudin or thrombin–3,4-DCI complex (each complex contained 0.5 U $ml^{-1}$ thrombin) for 30 min at 37°C, and the adhesion was tested after 60 min. In other experiments SW-480 cells were incubated with various concentrations of cycloheximide or actinomycin D for 20 min at room temperature before the addition of thrombin (0.5 U $ml^{-1}$) for 30 min at 37°C. Data are presented as mean ± s.e.m. ($n=4$). $^*P<0.05$, $^{**}P<0.01$, $^{***}P<0.001$, compared with thrombin-enhanced adhesion.

with the vitronectin ($\alpha_v\beta_3$) or fibronectin ($\alpha_5\beta_1$) receptors, resulting in marked inhibition in thrombin-enhanced adhesion to both human ECs and FN (Figure 4a–c respectively). However, in all cases, adhesion was reduced to basal levels by pretreatment with $7E_3$ (30 $\mu$g ml$^{-1}$), an MAb that recognises an antigenic determinant on the $\beta$ subunit of GpIIb/IIIa (i.e. GpIIIa) (Chopra *et al.*, 1988), suggesting that $\beta_3$ integrins played an important role in thrombin-enhanced SW-480 cell adhesion. Rhodostomin, an RGD-containing peptide, potently blocked the SW-480 cell adhesion response to thrombin at a concentration of 2.3 $\mu$g ml$^{-1}$ (0.3 $\mu$M). The synthetic peptide RGDS had a similar inhibition with the maximal effect at 75 $\mu$g ml$^{-1}$ (176 $\mu$M). On a molar basis, therefore, rhodostomin is about 600 times more potent than RGDS in inhibiting thrombin-enhanced adhesion.

### *Effect of rhodostomin on thrombin-enhanced SW-480 tumour cell migration*

The migratory activity of SW-480 cells was assayed in millicell-PCF inserts by using 24-well plates as described in Materials and methods. As shown in Figure 5, SW-480 human colon adenocarcinoma cells migrated positively to FN, vitronectin (VN) and laminin (LM) containing compartments. Pretreatment of SW-480 cells with thrombin (0.5 U ml$^{-1}$ for 30 min at 37°C) significantly enhanced migration to VN and FN, whereas it did not enhance SW-480 tumour cell migration to LM or control medium containing bovine serum albumin. Cells in the presence of rhodostomin (2.8 $\mu$g ml$^{-1}$) showed significant reduced activity in both control- and thrombin-enhanced migration (Figure 5), which were reduced approximately to the level of that in control medium. Likewise, the synthetic peptide RGDS at 80 $\mu$g ml$^{-1}$ had a similar inhibitory effect. On a molar basis, rhodostomin is about 500 times more potent than RGDS at inhibiting control- and thrombin-enhanced migration. These enhanced migrations were also reduced to basal levels by pretreatment with 30 $\mu$g ml$^{-1}$ $7E_3$ (data not shown).

### *Effect of thrombin on integrin expression in SW-480 cells*

Since MAb $7E_3$ completely blocked thrombin-enhanced adhesion on both ECs and FN, we examined the surface expression of $\beta_3$ integrins, the binding epitopes for $7E_3$, on SW-480 cells following stimulation with thrombin. Pretreatment of SW-480 cells with 0.5 U ml$^{-1}$ thrombin (30 min at 37°C) was followed by primary labelling with $7E_3$ and subsequently incubation with anti-mouse IgG-FITC. Fluorescence intensity was analysed by flow cytometry as described

**Figure 3** Effects of (**a**) staurosporine, and (**b**) calphostin C, on basal and thrombin-enhanced SW-480 tumour cell adhesion. SW-480 cells were preincubated with various concentrations of staurosporine or calphostin C for 20 min at room temperature, then stimulated with thrombin (0.5 U ml$^{-1}$) for 30 min at 37°C. Finally, these cells were washed once, resuspended and allowed to adhere to fibronectin-coated plates for 60 min at 37°C. TPA (0.1 $\mu$M, 37°C for 5 min) was used as a positive control for PKC activation, as we previously reported (Chiang *et al.*, 1994*b*). Data are presented as mean±s.e.m. ($n$=4–5). For (**a**), *$P<0.05$, **$P<0.01$, ***$P<0.001$; for (**b**), ##$P<0.01$, ###$P<0.001$. Both symbols are compared with respective thrombin-enhanced adhesion.

**Figure 4** Inhibition of thrombin-enhanced SW-480 cell adhesion by MAbs, rhodostomin and RGDS. SW-480 cells were stimulated with thrombin (0.5 U ml$^{-1}$) at 37°C for 30 min, then washed once and incubated with $10E_5$, $7E_3$ (both at 30 $\mu$g ml$^{-1}$), rhodostomin (2.3 $\mu$g ml$^{-1}$) or RGDS (75 $\mu$g ml$^{-1}$) for 30 min at room temperature. Finally, these cells were allowed to adhere to (**a**) HUVEC, (**b**) HMVEC and (**c**) fibronectin (FN)-coated plates for 60 min at 37°C. Data are presented as mean±s.e.m. ($n$=4–5).

in Materials and methods. As shown in Figure 6a and b, thrombin-stimulated cells exhibited an increase in the number of cells positively stained for $\beta_3$ integrins and an enhanced fluorescence. Figure 6c shows the quantitative results of $\beta_3$ integrin expression on SW-480 cells stimulated by thrombin, indicating that both mean fluorescence intensity and the mean number of positively staining cells were significantly increased. However, cells probed first with MAbs MAB1977 (anti-$\beta_1$ integrin), MCA698 (anti-$\alpha_5\beta_1$) or MCA699 (anti-$\alpha_6\beta_1$) did not show a significant increase in fluorescence intensity in response to thrombin pretreatment (data not shown).

### Discussion

Rhodostomin, an RGD-containing disintegrin, has been demonstrated to directly impair fibrinogen interaction with its specific receptor associated with GP IIb/IIIa (Huang *et al.*, 1987*b*, 1990). We previously showed that rhodostomin is about 18 000 times more potent that the synthetic peptide GRGDS in inhibiting SW-480 TCIPA, a thrombin-dependent reaction owing to SW-480 tissue factor activity expression, by virtue of its antiplatelet activity (Chiang *et al.*, 1994*a*). We also reported that rhodostomin binds via its RGD sequence to multiple integrin receptors (i.e. $\alpha_{IIb}\beta_3$, $\alpha_v\beta_3$, $\alpha_5\beta_1$) expressed on the SW-480 cell surface, and thereby blocks the adhesion of SW-480 cells to ECM (Chiang *et al.*, 1994*b*). In the present study, rhodostomin was further shown to significantly block thrombin-enhanced adhesion and migration of SW-480 cells, the two major events determining the tumour metastasis potential. Rhodostomin was about 600 and 500 times more potent than RGDS in inhibiting thrombin-enhanced SW-480 cell adhesion and migration respectively.

Activation of blood coagulation, a common feature in cancer patients, leads to the generation of thrombin. The biological effect of thrombin, as well as its role in TCIPA and fibrin formation may favour the metastatic spread of cancer by promoting cell migration, mediating tumour cell adhesion, stimulating autocrine growth factor secretion or inducing neovascularisation (Costantini and Zacharski, 1993; Wojtukiewicz *et al.*, 1992; Zacharski *et al.*, 1990). In the present study, thrombin was found to enhance SW-480 tumour cell adhesion and migration with the optimal concentration of 0.5 U $ml^{-1}$. According to a previous study, during clotting of whole blood or plasma, only approximately 15 U $ml^{-1}$

**Figure 5** Effect of thrombin on SW-480 cell migration and its inhibition by rhodostomin and RGDS. Cell migration assays were carried out in millicell-PCF inserts by using a standard 24-well tissue culture plates as described in Materials and methods. The lower compartment was filled with media containing fibronectin (FN), vitronectin (VN) and laminin (LM). Thrombin-treated SW-480 cells ($3 \times 10^5$ cells $ml^{-1}$) were added to the upper compartment of a millicell-PCF insert in the absence or in the presence of rhodostomin ($2.8 \mu g ml^{-1}$) or RGDS ($80 \mu g ml^{-1}$). Migration assays were performed for 14 h at 37°C in a humidified atmosphere containing 5% carbon dioxide. Data are presented as mean ± s.e.m. ($n=4$). *$P<0.05$, **$P<0.01$, ***$P<0.001$.

**Figure 6** Quantification by flow cytometry of thrombin-enhanced surface expression of $\beta_3$ integrins on SW-480 cells. SW-480 cells were pretreated with thrombin ($0.5 U ml^{-1}$, 37°C for 30 min) and then labelled with MAb $7E_3$ ($15 \mu g ml^{-1}$, room temperature for 60 min), followed by incubation with anti-mouse IgG-FITC. Mean fluorescence intensity was quantified on an EPICS computer system. Thrombin-treated cells (**b**) showed an increase in fluorescence intensity when compared with cells incubated with buffer (**a**). (**c**) Thrombin-treated SW-480 cells demonstrated increases in both the mean fluorescence intensity (□) and the mean number of positively staining cells (■) when compared with control cells incubated with buffer only. Data are presented as mean ± s.e.m. ($n=4$). *$P<0.05$, **$P<0.01$.

thrombin is ever measurable, despite more than 80% consumption of prothrombin (Fenton, 1988). Therefore, tumour cells require only low subclotting concentrations of thrombin to elicit their optimal adhesion response. One possibility leading to prolonged tumour cell exposure to low thrombin concentrations in the microcirculation is the interactions of tumour cells with platelets and blood plasma components, resulting in the formation of microthrombi containing tumour emboli. This unique clot structure may serve as a thrombin reservoir and protects thrombin from neutralisation by serine protease inhibitors (Fenton, 1988). Thus, tumour cells entrapped in the fibrin clot may be under a constant stimulation by thrombin and become more predisposed to successful metastasis.

The optimal effectiveness of low concentrations of thrombin for enhanced adhesion and migration suggests a thrombin-receptor-mediated mode of action. At present, there are two major thrombin receptors reported. Glycoprotein Ib (GP Ib), the predominant thrombin-binding molecule on human platelets, was implicated in both high- and moderate-affinity pathways of platelet activation (Greco and Jamieson, 1991). In our study, thrombin-enhanced adhesion was not significantly affected by pretreatment of SW-480 cells with $AP_1$ (25 $\mu$g ml$^{-1}$), the MAb of GP Ib (data not shown), indicating that the enhanced adhesion did not result from the binding of thrombin to GP Ib on tumour cells. The other candidate is a high-affinity functional thrombin receptor, a member of the superfamily of seven-pass transmembrane proteins (Vu *et al.*, 1991). mRNA encoding this receptor was detected in human platelets and ECs. Thrombin cleaves this receptor's amino-terminal extension to create a new receptor amino terminus that functions as a tethered ligand and activates the receptor (Vu *et al.*, 1991). Recently Wojtukiewicz *et al.* (1995) reported that thrombin effect is mediated through the 'tethered ligand' thrombin receptor expressed on human colon adenocarcinoma (clone A) as well as rat and mouse solid tumours, thereby promoting tumour cell adhesion to FN. Thus, we propose that the enhanced adhesive and migratory effects of thrombin on SW-480 human colon adenocarcinoma cells probably operate in a similar manner via binding to the 'tethered ligand' thrombin receptor on SW-480 cells. However, the exact entities that are responsible for thrombin-enhanced $\beta_3$ integrin expression need further investigation.

Our study on thrombin-enhanced SW-480 tumour cell adhesion to both ECs and FN revealed a bell-shaped dose–response curve. No stimulatory effect was observed when tumour cells were incubated with higher doses (i.e. 5 U ml$^{-1}$) of thrombin. At present, a precise interpretation of this dose dependency of the thrombin effect is unknown. One possibility is that thrombin-elicited effects on adhesion (probably through binding to its high-affinity receptors on tumour cells) are down-regulated (or desensitised) at high doses of agonist, a phenomenon that is characteristic of most G-protein-linked reactions (Greco and Jamieson, 1991; Wojtukiewicz *et al.*, 1993; Manning and Brass, 1991). An intact active site serine residue of thrombin molecule was a prerequisite for its effect on tumour cell adhesion at full potential since pretreatment of thrombin with hirudin or 3,4-DCI demonstrated a lower potency than the native form of the enzyme.

Adhesion of tumour cells to endothelium as well as ECM are partly mediated by the $\beta_3$ integrins (i.e. $\alpha_{IIb}\beta_3$ and $\alpha_v\beta_3$) expressed by these cells. Our laboratory previously reported that phorbol ester and a lipoxygenase metabolite of arachidonic acid (i.e. 12-(S)-HETE) specifically up-regulate SW-480 cell-surface expression of $\alpha_{IIb}\beta_3$ receptors (Chiang *et al.*, 1994*b*). In this study, we showed that thrombin challenged SW-480 cells, resulting in an increased fluorescence intensity of immunoreactive $\beta_3$ integrin probed by using MAb $7E_3$, which appears to recognise an antigenic determinant on the $\beta$ subunit of GpIIb/IIIa (i.e. GpIIIa) (Chopra *et al.*, 1988). However, $10E_5$, an MAb that does not cross-react with the integrins $\alpha_v\beta_3$ and $\alpha_5\beta_1$ (Grossi *et al.*, 1988), markedly blocked the thrombin-enhanced adhesion to ECs and FN. When compared with the inhibitory potency of $7E_3$, these results indicate that enhanced adhesion ought to involve mainly $\alpha_{IIb}\beta_3$ rather than $\alpha_v\beta_3$. On the other hand, the role of $\alpha_v\beta_3$ in the up-regulation ought not to be excluded as SW-480 cells showed enhanced migratory activity towards chemoattractant VN, the primary ligand for $\alpha_v\beta_3$. Similarly, the enhanced migration was also mediated by $\alpha_{IIb}\beta_3$, the predominant matrix receptor of platelets that mediates the binding of FN (Ruoslahti, 1991). This thrombin-enhanced surface expression of $\beta_3$ integrins did not require transcriptional regulation and *de novo* protein synthesis as it was not affected by actinomycin D and cycloheximide, which is consistent with the results from Nierodzik *et al.* (1992). In our previous study (Chiang *et al.*, 1994*b*), SW-480 cells possess an intracellular pool of integrin receptors, from which integrins translocate to the cell surface following PKC activation. Thus we propose that thrombin might also promote translocation of $\beta_3$ integrins from the intracellular pool to the plasma membrane, which may aid in adhesive and migratory properties of SW-480 cells. However, we do not exclude the additional possibility of affinity regulation of $\beta_3$ integrins by thrombin, since PKC activation has already been reported to cause an increase in the fibrinogen-binding affinity of $\alpha_{IIb}\beta_3$ in platelets (the 'inside-out' signalling) (O'Toole *et al.*, 1991; Du *et al.*, 1993). Such intracellular events may influence the conformation and binding affinity of the extracellular domain of integrins, thereby altering the strength and ligand preferences of cell adhesion (Ginsberg *et al.*, 1992; Brown 1988).

The mechanism whereby PKC activation translates into adhesive protein up-regulation and increased tumour cell adhesion remains to be elucidated. PKC represents a family of serine/threonine protein kinases that provide regulatory functions in intracellular signal transduction and are implicated in tumour growth, promotion and differentiation as well as oncogene activation and carcinogenesis (Liu *et al.*, 1992). Several observations (Grossi *et al.*, 1989; Chopra *et al.*, 1991) have indicated that 12(S)-HETE works via PKC activation to promote tumour cell adhesion to ECs. Likewise, PKC has been suggested to be involved in mediating the cellular effects of thrombin (Gomez *et al.*, 1988; di Corleto and de la Motte, 1989). Therefore, we used the non-selective but potent protein kinase inhibitor, staurosporine, and the selective inhibitor of PKC, calphostin C, to examine the effect of thrombin. Calphostin C markedly prevented thrombin-enhanced adhesion, which is similar to the effect of staurosporine. This indicates that thrombin-elicited effects probably operate via PKC activation.

In our study, thrombin challenge of human colon adenocarcinoma cells up-regulates cell-surface expression of $\beta_3$ integrins, which promote the migration and adhesion of tumour cells to FN and ECs. Because the interactions of tumour cells with platelets and plasma components generate thrombin and some lipoxygenase metabolite of arachidonic acid (i.e. the thrombin-dependent TCIPA owing to tissue factor activity expression on SW-480 cells), which may potentiate metastatic ability, our serial studies implied that if a significant number of colon adenocarcinoma cells induced TCIPA followed by thrombin-enhanced metastasis process, PKC inhibitors as well as integrin receptor antagonists (particularly the potent Arg-Gly-Asp-containing venom peptide, rhodostomin) might be considered as possible adjuvant therapeutic agents in the prevention of certain cancer metastasis. Further studies are being undertaken to explore the antimetastatic effect of rhodostomin on the metastasis of SW-480 cells in experimental animals.

**Acknowledgements**

The authors would like to thank Miss Wen-Chun Chang for her excellent technical assistance. This work was financially supported by a grant from the National Science Council of Taiwan (NSC 81-0412-B002-114, NSC 83-0412-B002-037 and NSC 84-2331-B002-031).

### References

BROWN PJ. (1988). Phorbol ester stimulation of fibronectin-mediated cell adhesion. *Biochem. Biophys. Res. Commun.*, **155**, 603–607.

BRUHN HD AND ZURBORN KH. (1993). Influences of clotting factors (thrombin, factor XIII) and fibronectin on the growth of tumor cells and leukemic cells *in vitro*. *Blut*, **46**, 85–88.

CAVANAUGH PG, SLOANE BF AND HONN KV. (1988). Role of the coagulation system in tumor cell-induced platelet aggregation and metastasis. *Haemostasis*, **18**, 37–46.

CHIANG HS, SWAIM MW AND HUANG TF. (1994*a*). Characterization of platelet aggregation induced by human colon adenocarcinoma cells and its inhibition by snake venom peptides, trigramin and rhodostomin. *Br. J. Haematol.*, **87**, 325–331.

CHIANG HS, PENG HC AND HUANG TF. (1994*b*). Characterization of integrin expression and regulation on SW-480 human colon adenocarcinoma cells and the effect of rhodostomin on basal and upregulated tumor cell adhesion. *Biochim. Biophys. Acta.*, **1224**, 506–516.

CHOPRA H, HATFIELD JS, CHANG YS, GROSSI IM, FITZGERALD LA, O'GARA CY, MARNETT LJ, DIGLIO GA, TAYLOR JD AND HONN KV. (1988). Role of tumor cell cytoskeleton and membrane glycoprotein IRGpIIb/IIIa in platelet adhesion to tumor cell membrane and tumor cell induced platelet aggregation. *Cancer Res.*, **48**, 3787–3800.

CHOPRA H, TIMAR J, CHEN YQ, RONG X, GROSSI IM, FITZGERALD LA, TAYLOR JD AND HONN KV. (1991). The lipoxygenase metabolite 12(S)-HETE indices a cytoskeleton-dependent increase in surface expression of integrin $\alpha_{IIb}\beta_3$ on melanoma cells. *Int. J. Cancer*, **49**, 774–786.

COSTANTINI V AND ZACHARSKI LR. (1993). Fibrin and cancer. *Thromb. Haemost.*, **69**, 406–414.

DI CORLETO PE AND DE LA MOTTE CA. (1989). Thrombin causes increased monocytic-cell adhesion to endothelial cells through a protein kinase C-dependent pathway. *Biochem. J.*, **264**, 71–77.

DU X, GU M, WEISEL JW, NAGASWAMI C, BENNETT JS, BOWDITCH R AND GINSBERG MH. (1993). Long range propagation of conformational changes in integrin $\alpha_{IIb}\beta_3$. *J. Biol. Chem.*, **268**, 23087–23092.

FENTON JW. (1988). Regulation of thrombin generation and functions. *Semin. Thromb. Hemost.*, **14**, 234–240.

FITZGERALD LA AND PHILLIPS DR. (1985). Calcium requirement of the platelet membrane glycoprotein IIb/IIIa complex. *J. Biol. Chem.*, **260**, 11366–11374.

GINSBERG MH, DU X AND PLOW EF. (1992). Inside-out integrin signalling. *Curr. Opin. Cell Biol.*, **4**, 766–771.

GOMEZ ML, MEDRANO EE, CAFFERATTA EGA AND TELLEZINON MT. (1988). Protein kinase C is differentially regulated by thrombin, insulin, and epidermal growth factor in human mammary tumor cells. *Exp. Cell Res.*, **175**, 74–80.

GRECO NJ AND JAMIESON GA. (1991). High and moderate affinity pathways for α-thrombin-induced platelet activation. *Proc. Soc. Exp. Biol. Med.*, **198**, 792–799.

GROSSI IM, HATFIELD JS, FITZGERALD LA, NEWCOMBE M, TAYLOR JD AND HONN KV. (1988). Role of tumor cell glycoproteins immunologically related to glycoproteins Ib and IIb/IIIa in tumor cell-platelet and tumor cell-matrix interactions. *FASEB J.*, **2**, 2385–2395.

GROSSI IM, FITZGERALD LA, UMBARGER LA, NELSON KK, GIGLIO CA, TAYLOR JD AND HONN KV. (1989). Bidirectional control of membrane expression and/or activation of the tumor cell IRGpIIb/IIIa receptor and tumor cell adhesion by lipoxygenase products of arachidonic acid and linoleic acid. *Cancer Res.*, **49**, 1029–1037.

HONN KV AND SLOANE BF. (1984). *Hemostatic mechanisms and metastasis*. Martinus Nijhoff: Boston.

HONN KV, TANG DG AND CHEN YQ. (1992*a*). Adhesion molecules and site-specific metastasis. In *Thrombosis: an update*. NeriSerneri GG, Gensini GF, Abbate R and Prisco D. (eds.), pp. 269–303. Scientific Press: Florence.

HONN KV, CHEN YQ, TIMAR J, ONODA JM, HATFIELD JS, FLIGIEL SEG, STEINERT BW, DIGLIO CA, GROSSI IM, NELSON KK AND TAYLOR JD. (1992*b*). $\alpha_{IIb}\beta_3$ integrin expression and function in subpopulations of murine tumors. *Exp. Cell Res.*, **201**, 23–32.

HUANG TF, HOLT JC, LAKASIEWICZ H AND NIEWIAROWSKI S. (1987*a*). Trigramin, a low molecular weight peptide inhibiting fibrinogen interaction with platelet receptors expressed on glycoprotein IIb–IIIa complex. *J. Biol. Chem.*, **262**, 16157–16163.

HUANG TF, WU YG AND OUYANG C. (1987*b*). Characterization of a potent platelet aggregation inhibitor from *Agkistrodon rhodostoma* snake venom. *Biochim. Biophys. Acta.*, **925**, 248–257.

HUANG TF, HOLT JC, KIRKY EPH AND NIEWIAROWSKI S. (1989). Trigramin: primary structure and its inhibition of von Willebrand Factor binding to glycoprotein IIb-IIIa complex on human platelet. *Biochemistry*, **28**, 661–666.

HUANG TF, OUYANG C AND TENG CM. (1990). XIth International Congress on Thrombosis, (Abstract 141). Ljubljana, Yugoslavia.

HUANG TF, WANG WJ, TENG CM AND OUYANG C. (1991*a*). Mechanism of action of the antiplatelet peptide, antin, from *Bitis arietans* venom. *Biochim. Biophys. Acta.*, **1074**, 144–150.

HUANG TF, LIU CZ, OUYANG C AND TENG CM. (1991*b*). Halysin, an Arg-Gly-Asp-containing peptide, inhibits platelet aggregation by acting as fibrinogen receptor antagonist. *Biochem. Pharmacol.*, **42**, 1209–1219.

JAFFE EA, NACHMAN RL, BECKER CG AND MINNICK CR. (1973). Culture of human endothelial cells derived from umbilical veins. *J. Clin. Invest.*, **52**, 2745–2756.

LIU B, RENAUD C, NELSON KK, CHEN YQ, BAZAZ R, KOWYNIA J, TIMAR J, DIGLIO CA AND HONN KV. (1992). Protein kinase C-inhibitor calphostin C reduces B16 amelanotic melanoma cell adhesion to endothelium and lung colonization. *Int. J. Cancer*, **52**, 147–152.

MCGREGOR BC, MCGREGOR JL, WEISS LM, WOOD GS, CHUNG-HONG HU, BOUKERCHE H AND WARNKE RA. (1989). Presence of cytoadhesins (IIb-IIIa-like glycoproteins) on human metastatic melanomas but not on benign melanocytes. *Am. J. Clin. Pathol.*, **92**, 495–499.

MANNING DR AND BRASS LF. (1991). The role of GTP-binding proteins in platelet activation. *Thromb. Haemost.*, **66**, 393–399.

MEDRANO EE, CAFFERATA EGA AND LARCHER F. (1987). Role of thrombin in the proliferative response of T-47D mammary tumor cells. *Exp. Cell Res.*, **172**, 354–364.

NIERODZIK MLR, KAJUMO F AND KARPATKIN S. (1992). Effect of thrombin treatment of tumor cells on adhesion of tumor cells to platelets in vitro and tumor metastasis in vivo. *Cancer Res.*, **52**, 3267–3272.

O'TOOLE TE, MANDELMAN D, FORSYTH J, SHATTIL SJ, PLOW EF AND GINSBERG MH. (1991). Modulation of the affinity of integrin $\alpha_{IIb}\beta_3$ (GPIIb-IIIa) by the cytoplasmic domain of $\alpha_{IIb}$. *Science*, **254**, 845–847.

RICKLES FR, LEVINE M AND EDWARDS RL. (1992). Hemostatic alterations in cancer patients. *Cancer. Metast. Rev.*, **11**, 237–248.

RUCINSKI B, NIEWIAROWSKI S, HOLT JC, SOSEKA T AND KNUDSEN KA. (1990). Batroxostatin, an Arg-Gly-Asp-containing peptide from *Bothrops atrox*, is a potent inhibitor of platelet aggregation and cell interaction with fibronectin. *Biochim. Biophys. Acta.*, **1054**, 257–262.

RUOSLAHTI E. (1991). Integrins. *J. Clin. Invest.*, **87**, 1–5.

SHEBUSKI RJ, RAMJIT DR, BENCEN GH AND POLOKOFF MA. (1989). Characterization and platelet inhibitory activity of Bitistatin, a potent Arginine-Glycine-Aspartic acid-containing peptide from the venom of the viper *Bitis arietans*. *J. Biol. Chem.*, **264**, 21550–21556.

VAPORCIYAN AA, JONES ML AND WARD PA. (1993). Rapid analysis of leukocyte-endothelial adhesion. *J. Immunol. Method*, **159**, 93–100.

VU T-KH, HUNG DT, WHEATON VI AND COUGHLIN SR. (1991). Molecular cloning of a functional thrombin receptor reveals a novel proteolytic mechanism of receptor activation. *Cell*, **64**, 1057–1068.

WOJTUKIEWICZ MZ, TANG DG, NELSON KK, WALZ DA, DIGLIO CA AND HONN KV. (1992). Thrombin enhances tumor cell adhesive and metastatic properties via increased $\alpha_{IIb}\beta_3$ expression on the cell surface. *Thromb. Res.*, **68**, 233–245.

WOJTUKIEWICZ MZ, TANG DG, CIARELLI JJ, NELSON KK, WALZ DA, DIGLIO CA, MAMMEN EF AND HONN KV. (1993). Thrombin increases the metastatic potential of tumor cells. *Int. J. Cancer*, **54**, 793–806.

WOJTUKIEWICZ MZ, TANG DG, BEN-JOSEF E, RENAUD C, WALZ DA AND HONN KV. (1995). Solid tumor cells express functional 'tethered ligand' thrombin receptor. *Cancer Res.*, **55**, 698–704.

ZACHARSKI LR, COSTANTINI V, WOJTUKIEWICZ MZ, MEMOLI VA AND KUDRYK J. (1990). Anticoagulants as cancer therapy. *Semin. Oncol.*, **17**, 217–227.

ZACHARSKI LR, MEEHAN KR, ALGARRA SM AND CALVO FA. (1992*a*). Clinical trials with anticoagulant and antiplatelet therapies. *Cancer Metast. Rev.*, **11**, 421–431.

ZACHARSKI LR, WOJTUKIEWICZ MZ, COSTANTINI V, ORNSTEIN DL AND MEMOLI VA. (1992*b*). Pathways of coagulation/fibrinolysis activation in malignancy. *Semin. Thromb. Hemost.*, **18**, 104–116.


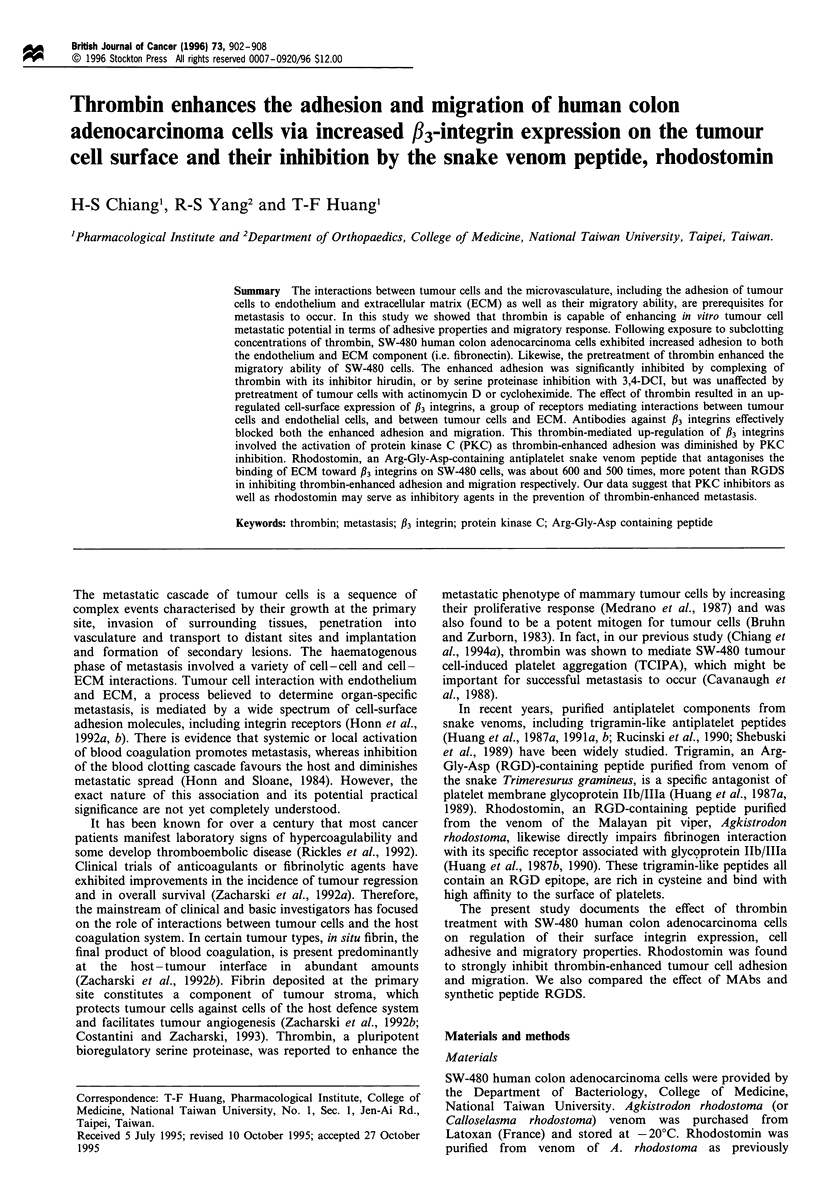

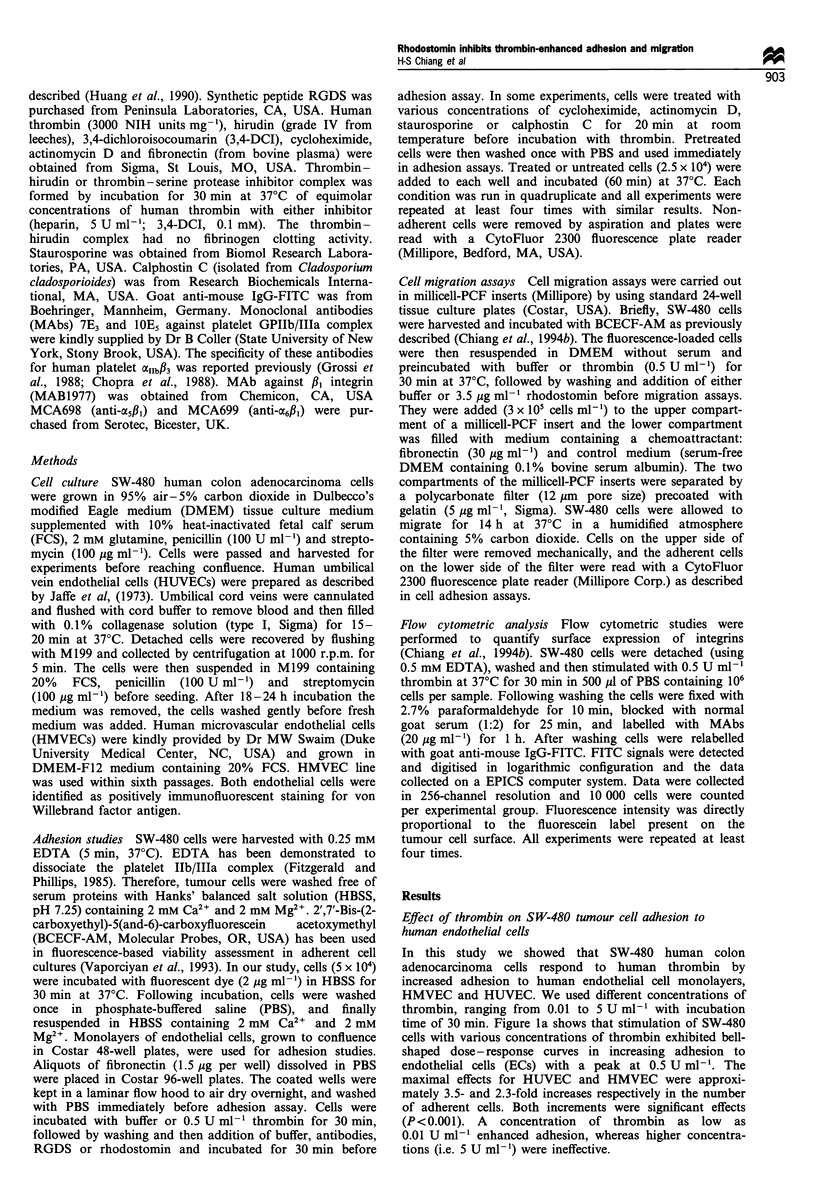

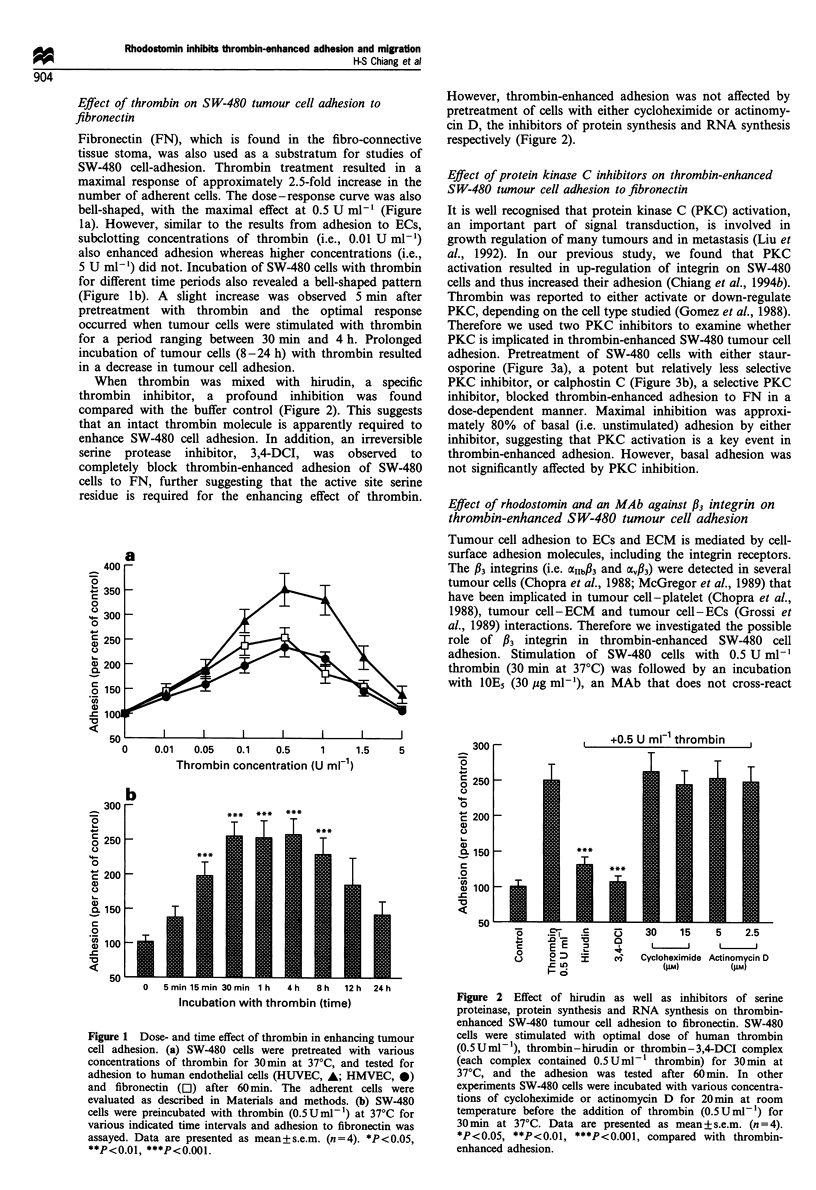

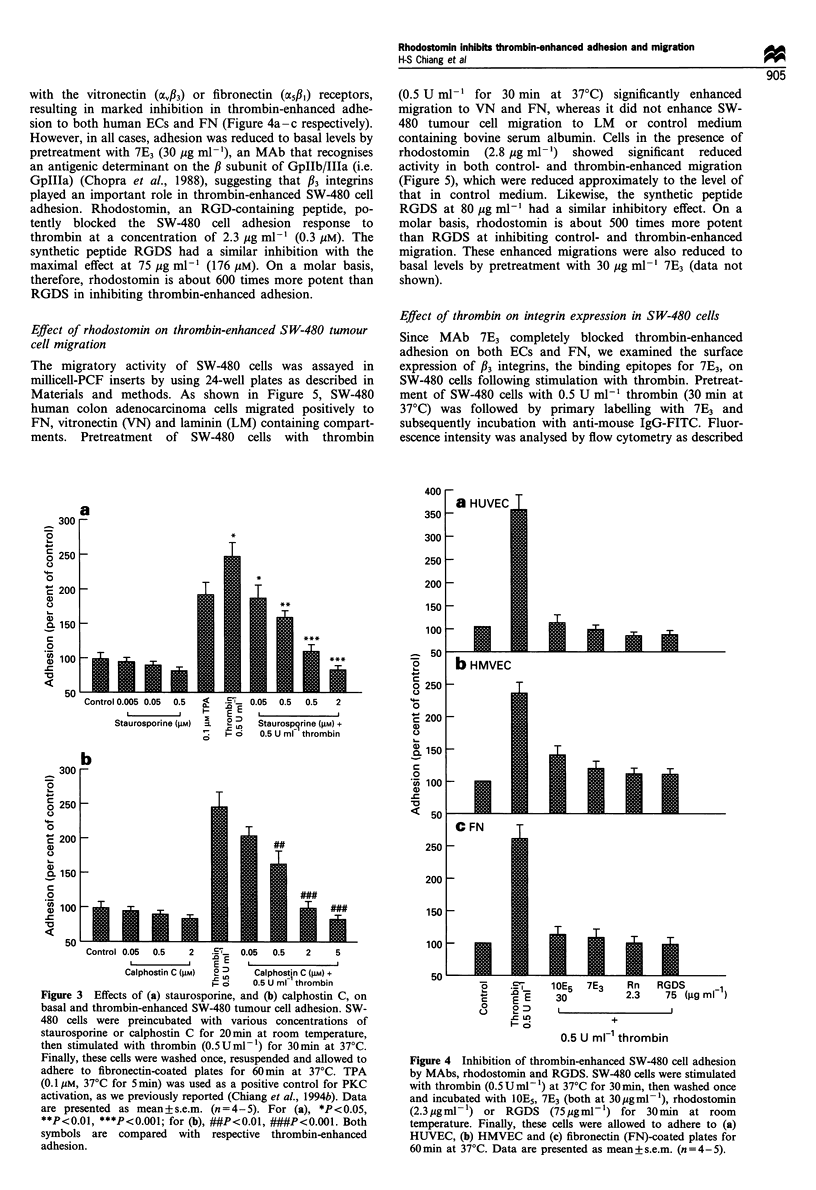

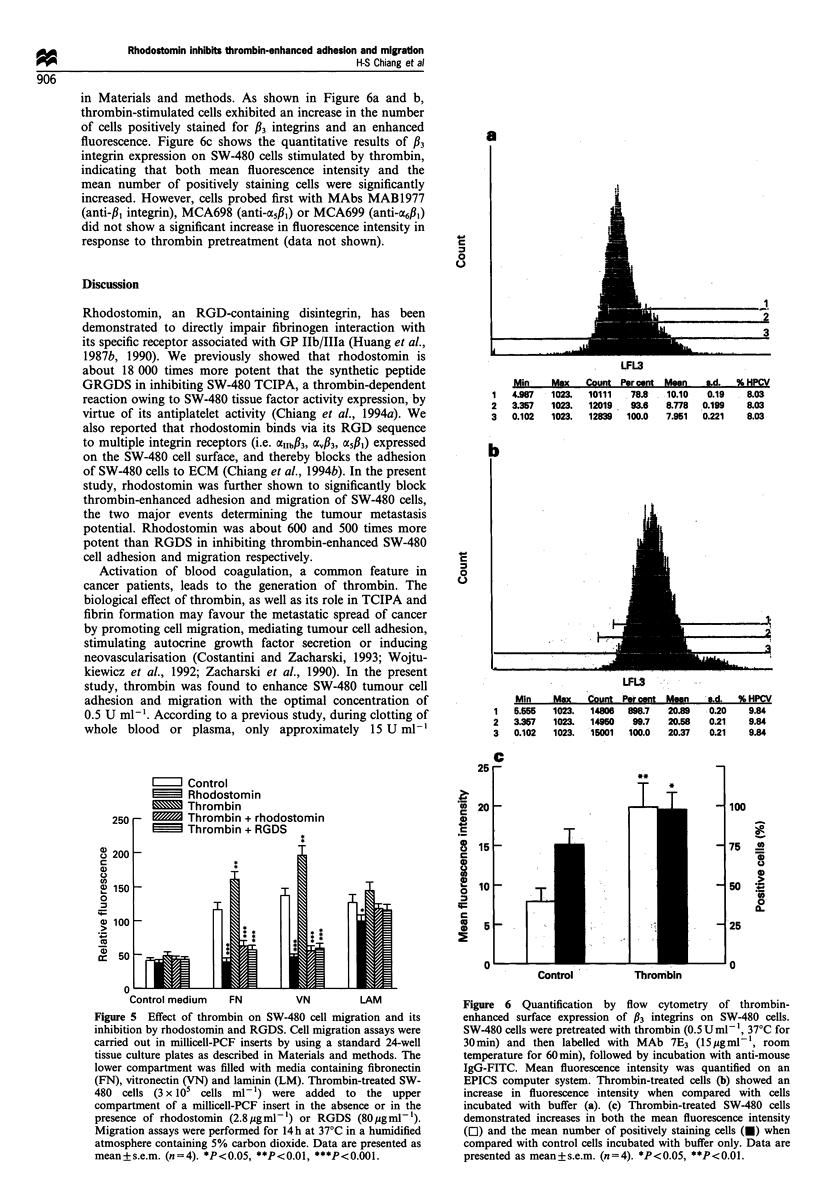

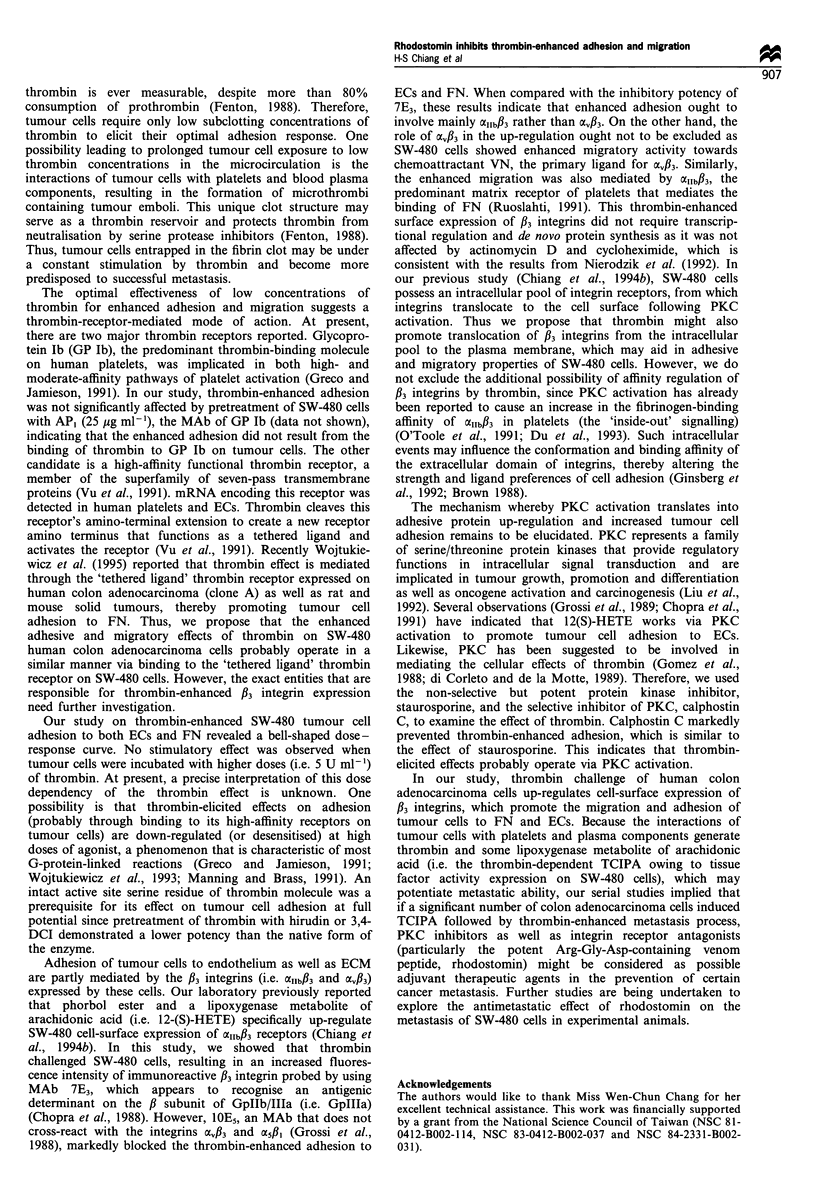

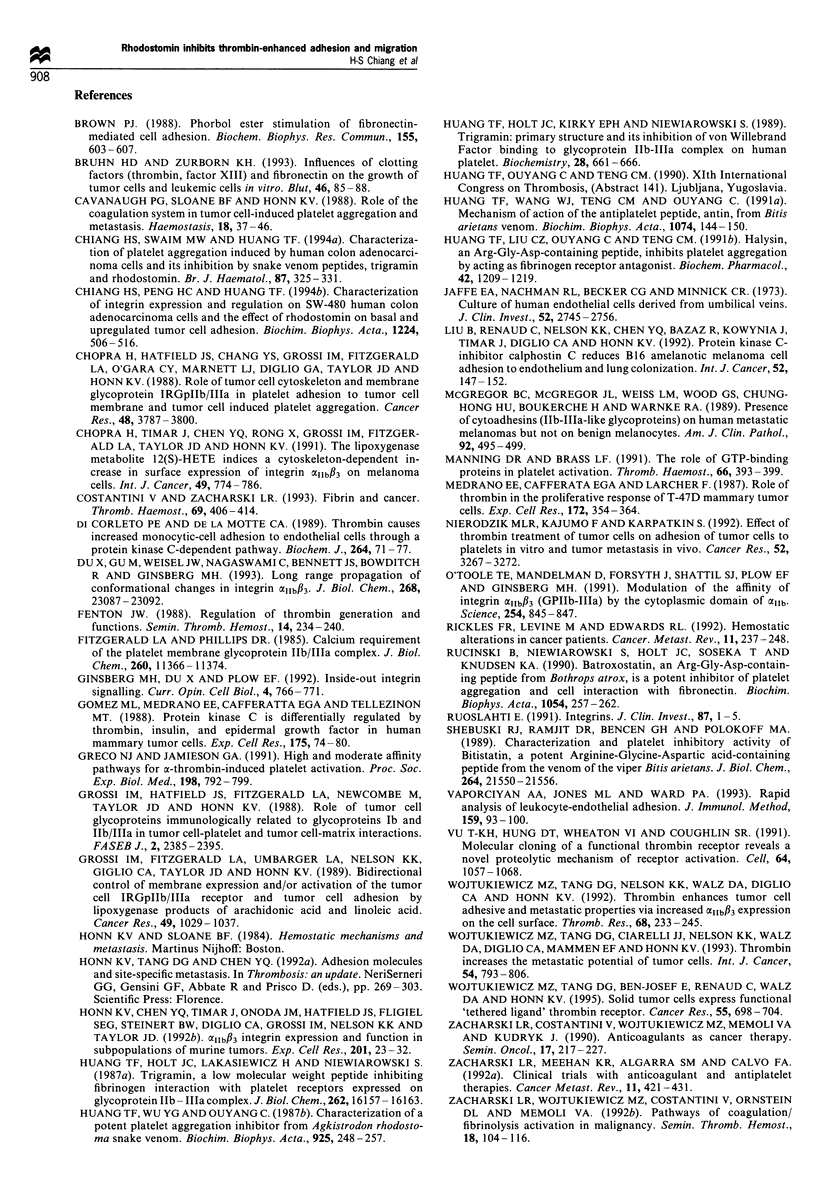


## References

[OCR_01024] Brown P. J. (1988). Phorbol ester stimulation of fibronectin-mediated cell adhesion.. Biochem Biophys Res Commun.

[OCR_01027] Bruhn H. D., Zurborn K. H. (1983). Influences of clotting factors (thrombin, factor XIII) and of fibronectin on the growth of tumor cells and leukemic cells in vitro.. Blut.

[OCR_01034] Cavanaugh P. G., Sloane B. F., Honn K. V. (1988). Role of the coagulation system in tumor-cell-induced platelet aggregation and metastasis.. Haemostasis.

[OCR_01045] Chiang H. S., Peng H. C., Huang T. F. (1994). Characterization of integrin expression and regulation on SW-480 human colon adenocarcinoma cells and the effect of rhodostomin on basal and upregulated tumor cell adhesion.. Biochim Biophys Acta.

[OCR_01039] Chiang H. S., Swaim M. W., Huang T. F. (1994). Characterization of platelet aggregation induced by human colon adenocarcinoma cells and its inhibition by snake venom peptides, trigramin and rhodostomin.. Br J Haematol.

[OCR_01053] Chopra H., Hatfield J. S., Chang Y. S., Grossi I. M., Fitzgerald L. A., O'Gara C. Y., Marnett L. J., Diglio C. A., Taylor J. D., Honn K. V. (1988). Role of tumor cytoskeleton and membrane glycoprotein IRGpIIb/IIIa in platelet adhesion to tumor cell membrane and tumor cell-induced platelet aggregation.. Cancer Res.

[OCR_01058] Chopra H., Timar J., Chen Y. Q., Rong X. H., Grossi I. M., Fitzgerald L. A., Taylor J. D., Honn K. V. (1991). The lipoxygenase metabolite 12(S)-HETE induces a cytoskeleton-dependent increase in surface expression of integrin alpha IIb beta 3 on melanoma cells.. Int J Cancer.

[OCR_01067] Costantini V., Zacharski L. R. (1993). Fibrin and cancer.. Thromb Haemost.

[OCR_01071] DiCorleto P. E., de la Motte C. A. (1989). Thrombin causes increased monocytic-cell adhesion to endothelial cells through a protein kinase C-dependent pathway.. Biochem J.

[OCR_01077] Du X., Gu M., Weisel J. W., Nagaswami C., Bennett J. S., Bowditch R., Ginsberg M. H. (1993). Long range propagation of conformational changes in integrin alpha IIb beta 3.. J Biol Chem.

[OCR_01082] Fenton J. W. (1988). Regulation of thrombin generation and functions.. Semin Thromb Hemost.

[OCR_01086] Fitzgerald L. A., Phillips D. R. (1985). Calcium regulation of the platelet membrane glycoprotein IIb-IIIa complex.. J Biol Chem.

[OCR_01091] Ginsberg M. H., Du X., Plow E. F. (1992). Inside-out integrin signalling.. Curr Opin Cell Biol.

[OCR_01095] Gomez M. L., Medrano E. E., Cafferatta E. G., Tellez-Inon M. T. (1988). Protein kinase C is differentially regulated by thrombin, insulin, and epidermal growth factor in human mammary tumor cells.. Exp Cell Res.

[OCR_01101] Greco N. J., Jamieson G. A. (1991). High and moderate affinity pathways for alpha-thrombin-induced platelet activation.. Proc Soc Exp Biol Med.

[OCR_01113] Grossi I. M., Fitzgerald L. A., Umbarger L. A., Nelson K. K., Diglio C. A., Taylor J. D., Honn K. V. (1989). Bidirectional control of membrane expression and/or activation of the tumor cell IRGpIIb/IIIa receptor and tumor cell adhesion by lipoxygenase products of arachidonic acid and linoleic acid.. Cancer Res.

[OCR_01107] Grossi I. M., Hatfield J. S., Fitzgerald L. A., Newcombe M., Taylor J. D., Honn K. V. (1988). Role of tumor cell glycoproteins immunologically related to glycoproteins Ib and IIb/IIIa in tumor cell-platelet and tumor cell-matrix interactions.. FASEB J.

[OCR_01129] Honn K. V., Chen Y. Q., Timar J., Onoda J. M., Hatfield J. S., Fligiel S. E., Steinert B. W., Diglio C. A., Grossi I. M., Nelson K. K. (1992). Alpha IIb beta 3 integrin expression and function in subpopulations of murine tumors.. Exp Cell Res.

[OCR_01145] Huang T. F., Holt J. C., Kirby E. P., Niewiarowski S. (1989). Trigramin: primary structure and its inhibition of von Willebrand factor binding to glycoprotein IIb/IIIa complex on human platelets.. Biochemistry.

[OCR_01135] Huang T. F., Holt J. C., Lukasiewicz H., Niewiarowski S. (1987). Trigramin. A low molecular weight peptide inhibiting fibrinogen interaction with platelet receptors expressed on glycoprotein IIb-IIIa complex.. J Biol Chem.

[OCR_01161] Huang T. F., Liu C. Z., Ouyang C. H., Teng C. M. (1991). Halysin, an antiplatelet Arg-Gly-Asp-containing snake venom peptide, as fibrinogen receptor antagonist.. Biochem Pharmacol.

[OCR_01154] Huang T. F., Wang W. J., Teng C. M., Ouyang C. (1991). Mechanism of action of the antiplatelet peptide, arietin, from Bitis arietans venom.. Biochim Biophys Acta.

[OCR_01140] Huang T. F., Wu Y. J., Ouyang C. (1987). Characterization of a potent platelet aggregation inhibitor from Agkistrodon rhodostoma snake venom.. Biochim Biophys Acta.

[OCR_01167] Jaffe E. A., Nachman R. L., Becker C. G., Minick C. R. (1973). Culture of human endothelial cells derived from umbilical veins. Identification by morphologic and immunologic criteria.. J Clin Invest.

[OCR_01170] Liu B., Renaud C., Nelson K. K., Chen Y. Q., Bazaz R., Kowynia J., Timar J., Diglio C. A., Honn K. V. (1992). Protein-kinase-C inhibitor calphostin C reduces B16 amelanotic melanoma cell adhesion to endothelium and lung colonization.. Int J Cancer.

[OCR_01186] Manning D. R., Brass L. F. (1991). The role of GTP-binding proteins in platelet activation.. Thromb Haemost.

[OCR_01177] McGregor B. C., McGregor J. L., Weiss L. M., Wood G. S., Hu C. H., Boukerche H., Warnke R. A. (1989). Presence of cytoadhesins (IIb-IIIa-like glycoproteins) on human metastatic melanomas but not on benign melanocytes.. Am J Clin Pathol.

[OCR_01190] Medrano E. E., Cafferata E. G., Larcher F. (1987). Role of thrombin in the proliferative response of T-47D mammary tumor cells. Mitogenic action and pleiotropic modifications induced together with epidermal growth factor and insulin.. Exp Cell Res.

[OCR_01195] Nierodzik M. L., Kajumo F., Karpatkin S. (1992). Effect of thrombin treatment of tumor cells on adhesion of tumor cells to platelets in vitro and tumor metastasis in vivo.. Cancer Res.

[OCR_01199] O'Toole T. E., Mandelman D., Forsyth J., Shattil S. J., Plow E. F., Ginsberg M. H. (1991). Modulation of the affinity of integrin alpha IIb beta 3 (GPIIb-IIIa) by the cytoplasmic domain of alpha IIb.. Science.

[OCR_01207] Rickles F. R., Levine M., Edwards R. L. (1992). Hemostatic alterations in cancer patients.. Cancer Metastasis Rev.

[OCR_01208] Rucinski B., Niewiarowski S., Holt J. C., Soszka T., Knudsen K. A. (1990). Batroxostatin, an Arg-Gly-Asp-containing peptide from Bothrops atrox, is a potent inhibitor of platelet aggregation and cell interaction with fibronectin.. Biochim Biophys Acta.

[OCR_01219] Shebuski R. J., Ramjit D. R., Bencen G. H., Polokoff M. A. (1989). Characterization and platelet inhibitory activity of bitistatin, a potent arginine-glycine-aspartic acid-containing peptide from the venom of the viper Bitis arietans.. J Biol Chem.

[OCR_01226] Vaporciyan A. A., Jones M. L., Ward P. A. (1993). Rapid analysis of leukocyte-endothelial adhesion.. J Immunol Methods.

[OCR_01231] Vu T. K., Hung D. T., Wheaton V. I., Coughlin S. R. (1991). Molecular cloning of a functional thrombin receptor reveals a novel proteolytic mechanism of receptor activation.. Cell.

[OCR_01250] Wojtukiewicz M. Z., Tang D. G., Ben-Josef E., Renaud C., Walz D. A., Honn K. V. (1995). Solid tumor cells express functional "tethered ligand" thrombin receptor.. Cancer Res.

[OCR_01241] Wojtukiewicz M. Z., Tang D. G., Ciarelli J. J., Nelson K. K., Walz D. A., Diglio C. A., Mammen E. F., Honn K. V. (1993). Thrombin increases the metastatic potential of tumor cells.. Int J Cancer.

[OCR_01238] Wojtukiewicz M. Z., Tang D. G., Nelson K. K., Walz D. A., Diglio C. A., Honn K. V. (1992). Thrombin enhances tumor cell adhesive and metastatic properties via increased alpha IIb beta 3 expression on the cell surface.. Thromb Res.

[OCR_01252] Zacharski L. R., Costantini V., Wojtukiewicz M. Z., Memoli V. A., Kudryk B. J. (1990). Anticoagulants as cancer therapy.. Semin Oncol.

[OCR_01257] Zacharski L. R., Meehan K. R., Algarra S. M., Calvo F. A. (1992). Clinical trials with anticoagulant and antiplatelet therapies.. Cancer Metastasis Rev.

[OCR_01262] Zacharski L. R., Wojtukiewicz M. Z., Costantini V., Ornstein D. L., Memoli V. A. (1992). Pathways of coagulation/fibrinolysis activation in malignancy.. Semin Thromb Hemost.

